# Chronic pain self-management for older adults: a randomized controlled trial [ISRCTN11899548]

**DOI:** 10.1186/1471-2318-4-7

**Published:** 2004-07-30

**Authors:** Mary Ersek, Judith A Turner, Kevin C Cain, Carol A Kemp

**Affiliations:** 1 Pain Research Department, Swedish Medical Center, 550 16^th ^Ave, Providence Professional Building Suite 405, Seattle, WA 98122-5699, USA; 2 Department of Biobehavioral Nursing and Health Systems, University of Washington School of Nursing, Box 357266, Seattle, WA 98195-1406, USA; 3 Department of Psychiatry and Behavioral Sciences and Department of Rehabilitation Medicine, University of Washington School of Medicine, Box 356560, Seattle, WA 98195-6560, USA; 4 Office for Nursing Research, University of Washington School of Nursing, Box 357265, Seattle, WA 98195-7265, USA; 5 Department of Biostatistics, University of Washington School of Public Health and Community Medicine, Box 357232, Seattle, WA 98195-7232, USA

## Abstract

**Background:**

Chronic pain is a common and frequently disabling problem in older adults. Clinical guidelines emphasize the need to use multimodal therapies to manage persistent pain in this population. Pain self-management training is a multimodal therapy that has been found to be effective in young to middle-aged adult samples. This training includes education about pain as well as instruction and practice in several management techniques, including relaxation, physical exercise, modification of negative thoughts, and goal setting. Few studies have examined the effectiveness of this therapy in older adult samples.

**Methods/Design:**

This is a randomized, controlled trial to assess the effectiveness of a pain self-management training group intervention, as compared with an education-only control condition. Participants are recruited from retirement communities in the Pacific Northwest of the United States and must be 65 years or older and experience persistent, noncancer pain that limits their activities. The primary outcome is physical disability, as measured by the Roland-Morris Disability Questionnaire. Secondary outcomes are depression (Geriatric Depression Scale), pain intensity (Brief Pain Inventory), and pain-related interference with activities (Brief Pain Inventory). Randomization occurs by facility to minimize cross-contamination between groups. The target sample size is 273 enrolled, which assuming a 20% attrition rate at 12 months, will provide us with 84% power to detect a moderate effect size of .50 for the primary outcome.

**Discussion:**

Few studies have investigated the effects of multimodal pain self-management training among older adults. This randomized controlled trial is designed to assess the efficacy of a pain self-management program that incorporates physical and psychosocial pain coping skills among adults in the mid-old to old-old range.

## Background

### The problem of chronic pain in the elderly

Chronic pain is a common problem in the elderly, and is often associated with significant physical disability and psychosocial problems [1]. Estimates of the prevalence of chronic pain problems among community-dwelling older adults range from 58–70% [1]. The most common painful conditions among older adults are musculoskeletal conditions such as osteoarthritis, low back pain, and previous fracture sites [2]. Chronic pain often results in depression, sleep disturbance, decreased mobility, increased health care utilization, and physical and social role dysfunction [1]. Despite its high prevalence, pain in the elderly often is inadequately assessed and treated [1].

As the United States population grows older, the public health problem of chronic pain and its sequelae will worsen. Projections show dramatic increases in this age group; approximately 25% of the population will be age 65 years or older in 2050. Moreover, by 2030 there will be an estimated 8 million people who are 85 years or older [3]. Thus, there is an urgent and growing need for interventions that are effective in decreasing pain, suffering, and pain-related disability in this group.

### The role of self-management in the treatment of chronic painful conditions

There is substantial empirical evidence that attention to cognitive and behavioral factors, in addition to physiological factors, is necessary for the successful treatment of chronic nonmalignant pain [4,5]. Empirically supported multimodal therapies that incorporate cognitive and behavioral strategies now exist for many chronic pain conditions, including rheumatoid arthritis, osteoarthritis, fibromyalgia, and low back pain [6-10]. These therapies aim to enhance the ability of patients to successfully self-manage their pain, using a variety of techniques. Related approaches and strategies are described under the rubrics "cognitive-behavioral therapy" (CBT), "psycho-educational" or "educational," and "self-management" or "self-help." Although there are variations among these approaches, they share some or all of the following components: education about pain, instruction in the identification and modification of negative thoughts, exercise, communication skills, relaxation training, and physical therapies. The goal of the therapies is to enhance function, improve mood, and decrease pain intensity by changing the emotional, cognitive, and behavioral responses to pain.

Despite their documented efficacy in young to middle-aged samples [9-12], cognitive-behavioral and self-management pain therapies have been little-studied in elderly populations. In one of the first examinations of CBT for elderly patients with pain, 69 outpatients with chronic pain were randomly assigned either to immediate treatment or delayed (wait list) treatment [13]. Approximately half of the sample was over 60 years of age, and age was unrelated to outcome. The intervention resulted in significant decreases in pain interference with daily activities and increases in participants' self-reported ability to cope with pain. Limitations of this study included the fairly small sample size and the lack of intent-to-treat analysis.

Keefe and colleagues [14] evaluated the efficacy of a pain coping skills training (CST) intervention as compared with arthritis education and standard care in decreasing pain and physical and psychological disability among 99 middle-aged to older outpatients with osteoarthritic knee pain. The CST consisted of 10 weekly group sessions focusing on identifying and reducing irrational thoughts, diverting attention away from the pain, and changing activity patterns to manage pain. The CST group showed significantly less pain and psychological disability following treatment as compared with the other two groups. At 6-month follow-up, the CST group showed significantly less physical and psychological disability as compared with the education group and marginally less psychological disability as compared with the standard care group [15]. Although this study provides evidence for the benefits of cognitive-behavioral therapy for older adults, it focused on arthritis patients and not older adults per se. Moreover, the average subject age was 64 years. It is not clear whether these findings would generalize to mid-old (i.e., 75–85 years) and old-old (85 years and older) adults. These groups have been shown to differ from their younger counterparts (i.e., those 65–74 years) in several dimensions, including pain prevalence, physical and cognitive function, involvement in recreational and social activities, and social support [16-19], that potentially could affect the pain experience and response to pain therapies.

One study that examined a cognitive-behavioral therapy in old-old adults (mean age 77.2 years) evaluated the efficacy of a 10-week CBT intervention (n = 11) versus an attention/support (AS) condition (n = 10) for nursing home residents [20]. The CBT condition incorporated pain education, progressive relaxation, imagery, coping skills training, cognitive restructuring, and attention diversion. CBT participants reported significantly less pain and pain-related disability following the intervention, as compared to the AS group. These significant differences were maintained at the 4-month follow-up. This study provides important evidence that CBT can be successfully applied to old-old adults; however, the results need to be replicated in other, larger samples, including non-institutionalized elderly.

### Retirement communities as a study setting

As the U.S. population continues to age, retirement communities have gained popularity. A retirement community allows older adults with varying lifestyles and physical abilities to live in an environment that encourages independence while providing needed access to health and social resources [21]. Although most residents of these communities live independently (some facilities also include assisted living apartments and skilled nursing facilities), the retirement community, on average, represents a mid-old to old-old population that is vulnerable to physical disability, health problems, and social isolation [22]. The growing population in retirement communities, then, is one in which self-management group therapies for chronic pain may hold great promise. Adoption of regular wellness-oriented pain management strategies may contribute to enhanced functioning and prolonged independence.

### Study purpose and specific aims

The primary goal of this study is to evaluate the efficacy of a pain self-management group intervention (SMG), as compared with a control condition (BOOK), in decreasing physical disability, pain, pain-related interference with activities, and depression in older retirement community residents with chronic pain. In addition, we wish to determine the extent to which SMG participation is associated with changes in specific pain-related beliefs and coping strategies, and the extent to which changes in these process variables are associated with changes in outcomes (physical disability, pain intensity, pain-related interference with activities, and depression). We plan to test the following hypotheses:

*1. *At post-treatment and each follow-up, participants assigned to SMG, as compared with participants assigned to BOOK, will report less physical disability (primary outcome), and lower pain intensity, pain-related interference with activities, and depressive symptom severity (secondary outcomes).

*2. *Participants assigned to SMG, as compared with participants assigned to BOOK, will show greater pre- to post-treatment increases in self-efficacy and use of adaptive pain coping strategies and greater decreases in catastrophizing. Significant differences between SMG and BOOK groups in pain-related beliefs and coping strategies will be maintained at 6-month and 1-year follow-ups.

*3. *Pre- to post-treatment changes in specific pain-related beliefs (catastrophizing, self-efficacy) and coping strategies (Chronic Pain Coping Inventory subscales) will be associated significantly with changes in physical and social functioning, pain intensity, and depression over the same period among SMG participants. These changes in beliefs and coping strategies will be maintained at 6-month and 1-year follow-ups.

**Figure 1:**
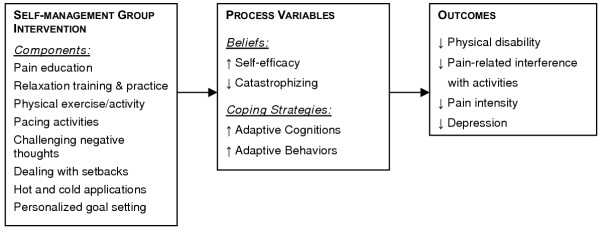
Hypothesized relationships among study variables

Figure 1 depicts the hypothesized relationships among study variables.

## Methods/Design

### Design

**Figure 2:**
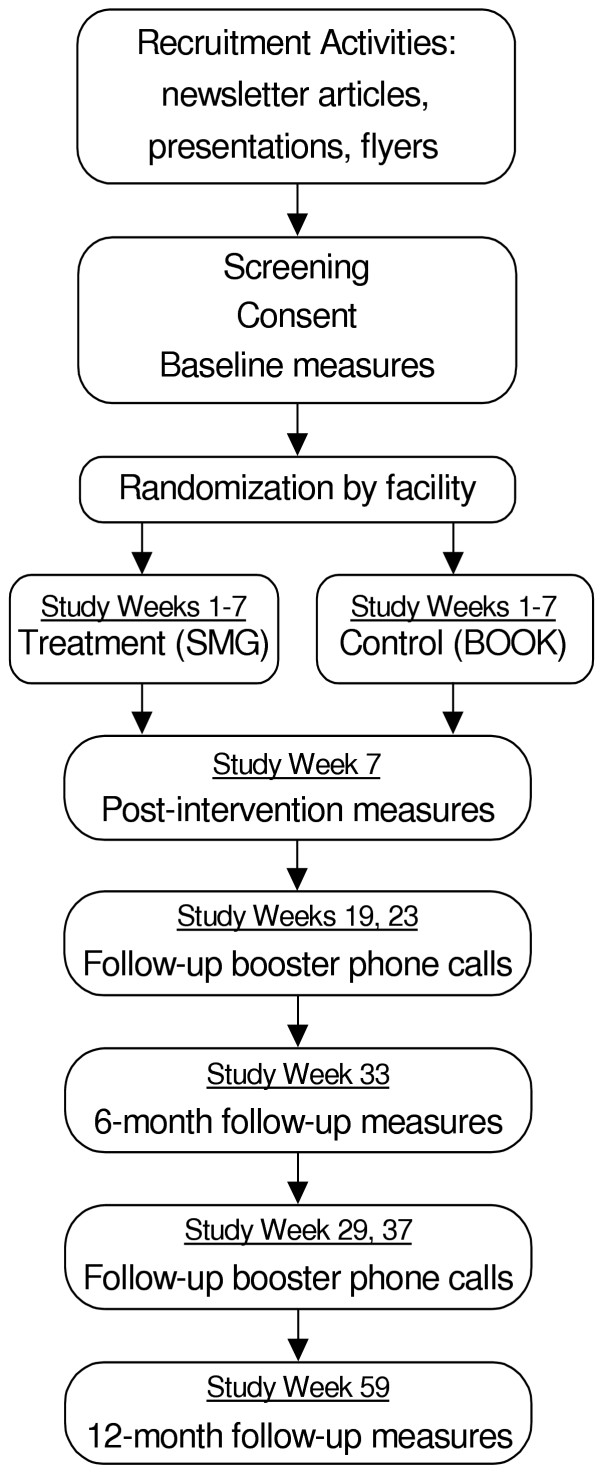
Study flowchart

This is a currently ongoing randomized controlled trial. The study procedures and measures have been approved by the Swedish Medical Center institutional review board. Figure 2 outlines study procedures and follow-up.

### Participants

Participants (targeted enrollment n = 273) are recruited from residents living in one of the 34 participating retirement communities in Seattle, Washington and the surrounding area. Study inclusion criteria are: (1) 65 years of age or older, (2) pain > 3 months duration that interferes with regular activities; and (3) ability to read and complete study questionnaires in English. Exclusion criteria are: (1) current, active cancer and (2) surgery within the past 6 months or surgery planned in the next 6 months.

### Recruitment and randomization procedures

Participants are recruited using newsletter announcements, flyers, brochures, and informational talks given at each facility. Retirement community residents who are interested in the study are screened to assess eligibility. Eligible residents who provide written informed consent are then asked to complete the baseline measures and to provide the name of their primary care provider (PCP), as well as permission to contact the PCP. PCPs of SMG group participants are sent a letter about the study and asked to contact the research nurse if there is any medical reason to restrict the resident's participation in the exercise portion of the study.

After all participants from a facility have completed the baseline questionnaires, the facility is randomized to receive either the BOOK or the SMG. Randomization is done by facility, rather than by individual participant, for several reasons. First, it expands the number of participating facilities by making feasible recruitment from smaller facilities. If approximately 5% of residents were recruited from any one facility, then it would not be scientifically or financially sound to involve facilities with fewer than 200 residents in independent or assisted living. A pilot study indicated that the ideal self-management group size is 5–12 participants. If we randomized within facilities, at least 10 participants would need to be recruited from each facility to allow 5 SMG participants. However, if all participants within a facility are randomized to the same condition, then smaller facilities can participate. If more than 12 residents in a facility randomized to the SMG condition enroll in the study, more than one group is scheduled. A second advantage of randomization by facility is that there is little risk of participants in one condition talking with participants randomized to another condition about their experiences in the study. Thus, there is less treatment contamination and likelihood that participants from different conditions will compare treatments, a situation that can provoke dissatisfaction among participants who do not receive the treatment of their choice.

### Pain self-management group (SMG)

The SMG intervention, which consists of seven weekly 90-minute group sessions, includes the major components of empirically-supported self-management interventions [23,24], refined for use with the elderly (see Table 1). For example, we include a discussion of myths about pain in older adults (e.g., pain is an inevitable part of aging) and focus on exercises that are effective and safe for older adults with musculoskeletal pain. The intervention is designed to decrease participants' physical disability and pain intensity; increase participation in home, social, and recreational activities; and enhance participants' self-efficacy for managing chronic pain. To accomplish these objectives, the intervention provides basic information about pain management, teaches problem-solving and relaxation skills, and provides practice with a variety of pain management techniques. Participants receive a class syllabus, relaxation tape, Theraband^® ^tubing for the performance of selected exercises, and two hot/cold gel packs.

**Table 1:** Summary of the self-management group intervention

**SESSION NUMBER**: TOPICS	MAJOR CONTENT AND ACTIVITIES
**Session 1**: Introduction; Basic principles of pain	Review purpose of the program/study. Review definition, types, & mechanisms of pain. Discuss myths about pain in older adults. Emphasize goals of chronic pain management. Discuss signs/symptoms that require medical attention. Introduce problem-solving techniques for pain management.
**Session 2**: Role of exercise & physical activity in pain management	Discuss exercise in pain management: problem of de-conditioning, types of exercise, tips for starting exercise program. Demonstrate & practice specific exercises. Introduce relaxation and breathing techniques as effective pain management strategies. Practice progressive muscle relaxation & abdominal breathing.
**Session 3**: Engaging in pleasant, meaningful activities; pacing activities	Discuss ways in which chronic pain may be limiting participation in enjoyable or meaningful activities Use problem solving to develop individualized plans for increasing these activities. Discuss strategies for activity pacing and rationale for avoiding guarding and inactivity. Practice relaxation.
**Session 4**: Challenging negative thoughts; Dealing with pain flare-ups and setbacks	Discuss critical role of thoughts and appraisals about pain in determining affective and behavioral responses to pain. Help participants to identify negative thoughts that they may have in response to pain. Practice challenging negative thoughts with positive thoughts about effective ways to manage pain. Discuss strategies for dealing with setbacks and pain-flare-ups. Practice relaxation.
**Session 5**: Non-drug pain therapies; Heat & cold; Dealing with pain flare-ups and setbacks (continued)	Describe rationale for using nondrug pain therapies. Describe and practice application of heat and cold; review precautions in using heat and cold for pain Continue discussion about coping with pain flare-ups & setbacks in pain management. Practice relaxation.
**Session 6**: Pain medications & complementary therapies	Describe the role of medications for pain management. Discuss the major types of pain medications. Describe the use of complementary therapies in pain management. Discuss steps in making informed decisions about all pain therapies.
**Session 7**: Pain management plan; Wrap-up	Discuss maintenance of gains made through the program. Review coping with set backs & pain flare-ups. Revise written individualized maintenance plans for each participant.

A key component of this self-management group is the development of personalized pain management plans. Participants begin developing a plan during the first class and revise it each week as they learn and practice additional pain management skills. With the assistance of the facilitator and, at times, other group members, participants review pain control strategies that they have learned and practiced and choose one or several strategies that best meet their individual needs and interests. Participants identify specifically what they will do (in measurable terms) and define the parameters (e.g., how many times per week, how far they will walk, how many repetitions of each exercise they will do). Although each person develops his or her own plan, the plans incorporate the same repertoire of activities that are taught in the class. These plans are monitored weekly during the classes and during follow-up phone calls (described below).

### Educational book control condition (BOOK)

Participants who are assigned to the BOOK condition receive a copy of *The Chronic Pain Workbook, 2*^*nd*^*Edition *[25]. Facilitators telephone participants 1 and 4 weeks after participants receive the workbook. The BOOK condition was designed to control for attention and information. In these calls, facilitators inquire about participants' current pain and functioning, and ask about use of pain therapies and self-management techniques. There is no specific therapeutic component in the phone calls and facilitators do not help BOOK participants identify goals or develop a pain management plan.

### Booster and follow-up phone calls

The SMG group facilitator telephones each participant at 12, 16, 22, and 30 weeks after the final group session. During the booster phone calls, facilitators inquire about pain and functioning, current pain management plans, and successes and obstacles in meeting pain management goals, as well as provide encouragement and assistance in problem-solving obstacles encountered in pain management. BOOK participants receive follow-up phone calls at the same intervals to control for attention.

### Steps taken to ensure and monitor group facilitator adherence

Group facilitators are nurses and psychologists with expertise in geriatrics and/or pain management and experience in facilitating therapeutic groups. All are specifically trained according to the treatment protocol. We monitor group facilitator adherence to the self-management group protocol, as recommended by Waltz et al. [26]. All facilitators receive and review a facilitator's syllabus that contains a detailed protocol describing the goals, contents, and activities for each of the 7 sessions. Group facilitators have met 3 times to discuss protocol and treatment integrity issues. Finally, each session for each treatment group is audiotaped. Twenty percent of the audiotapes are randomly chosen and reviewed by a trained research nurse who is not involved in any other aspects of the study. The research nurse listens to the tapes and evaluates the degree to which the group sessions are conducted according to the protocol using a checklist developed for this purpose.

### Measures

Study measures were chosen based on psychometric properties, including sensitivity to change; brevity; and appropriateness for use with community-dwelling, older adults with chronic pain. They are described below and summarized in Table 2.

**Table 2:** Measures and assessment times

**CONSTRUCT/MEASURE**	**SCREENING/BASELINE**	**POST-INTERVENTION**	**6-MONTH FOLLOW-UP**	**12-MONTH FOLLOW-UP**
**Physical Functioning**	√	√	√	√
Roland-Morris Disability Questionnaire				
Brief Pain Inventory (BPI) – pain interference subscale				
**Pain Intensity**	√	√	√	√
Brief Pain Inventory – pain intensity subscale				
**Mood Disturbance/ Social functioning**	√	√		√
Geriatric Depression Scale				
**Pain Beliefs and Coping**	√	√		√
Chronic Pain Coping Inventory – (*includes pain medication use)*				
Coping Strategies Questionnaire – catastrophizing, praying/hoping subscales				
Self-efficacy Scale				
**Pain Knowledge**	√	√		
**Demographics, Medical Conditions, Medications**	√			
Screening & Intake Questionnaire				
Adapted Charlson Index				
**Cognitive Functioning**	√			
Folstein Mini-Mental State Examination				
**Pretreatment Expectations**	√			
**Adherence to Treatment**		√		√
Attendance at classes				
Completion of reading assignments				
Attainment of goals (Personal Pain Management Plan)				

#### Descriptive measures

The following measures are administered at baseline to describe the sample and to explore whether these variables are associated with treatment response. We will compare the two study groups on these measures to determine whether they are comparable at baseline.

##### Screening and intake interview schedule – demographic information and pain history

During the screening process and baseline assessment, participants are asked a series of questions to elicit demographic and pain history variables, including age, race, ethnicity, gender, marital status, education level, sites and duration of pain, and prior and current pain treatments.

##### Folstein mini-mental state examination (MMSE) [27]

The MMSE is a measure that is widely used to assess cognitive function, particularly in older adults. It consists of 30 items, and requires 5–10 minutes to administer. Items assess orientation, memory, attention, and calculation. The MMSE has been demonstrated to be valid and to have good test-retest reliability [28].

##### Charlson index of comorbidity (CI)

The CI is an extensively used, valid, and reliable measure of comorbid medical conditions [29]. The CI uses 19 categories of comorbidity; each category is weighted and scored according to an algorithm [29]. Higher scores indicate greater health burden from comorbid causes. In this study, we are using a self-report version of the CI demonstrated to be reliable and valid in a group of older adults [30]. Because comorbid conditions may be associated with pain appraisal, coping, and outcomes [31], we will examine the association between comorbid conditions and response to therapy.

#### Process measures

##### Self-efficacy scale (SES)

Participants complete the 8-item version of Lorig et al.'s Self- Efficacy Scale [32], which assesses confidence in ability to manage pain and associated problems such as fatigue and negative mood [33,34]. Previous studies have supported the reliability and validity of this measure [32,34,35]. The SES has been tested and used in studies of older adults [36].

##### Coping strategies questionnaire (CSQ) [37]

The CSQ is one of the most widely used measures of pain coping and catastrophizing [38,39]. Measures derived from the CSQ have been shown to be associated with various measures of functioning among patients with different pain conditions [38,40-43]. The CSQ has demonstrated reliability and validity in several samples of older adults, including those who are older than 75 years [44]. For this study, only the catastrophizing and praying/hoping subscales are used. Catastrophizing is included because prior studies have shown that this variable is associated with pain intensity, depression, and disability [45]. The praying/hoping subscale was included because this coping strategy has been found to be associated with the pain experience of older persons[46].

##### Chronic pain coping inventory (CPCI)

The CPCI measures cognitive and behavioral coping strategies used by people to manage chronic pain. It contains 9 subscales: guarding, resting, asking for assistance, relaxation, task persistence, exercise/stretch, seeking support, coping self-statements, and medication use [47]. The CPCI scales have been shown to have acceptable internal consistency and test-retest reliability, and to be associated significantly with physical disability and depression [47-49]. Additional development and psychometric testing have supported the reliability and validity of an additional activity pacing subscale [49].

##### Pretreatment expectations

Prior to learning the study condition to which they are randomized, participants are asked the degree to which they believe that each study condition will be helpful to them. They respond using a 0 to 10 scale, with 0 indicating "not helpful at all" and 10 indicating "extremely helpful."

##### Treatment adherence

1. Class attendance. Group leaders document weekly class attendance. Total attendance is assessed as a percentage of classes the participant attended (out of 7).

2. Reading log/usefulness. Both BOOK and SMG participants complete a form in which they report the amount read on each topic using a 0–5 scale ("I did not look at the section at all" to "I read the section thoroughly"). They also rate the usefulness of each section using a 0–5 scale ("Not at all useful" to "Very useful").

3. Goal attainment. Attainment of SMG participants' pain management goals is assessed using the Personal Pain Management Plan (PPMP). Each week, participants in the SMG group are asked to document the type and frequency of each activity they have chosen to utilize in the management of their chronic pain. They monitor and document the pain management activities that they actually performed over the week. Participants also document obstacles that they have encountered in trying to meet their goals and the solutions they have identified to overcome those obstacles. This form is printed on 2-page paper. The top copy is turned in each week and participants keep the bottom copy for their own records. The PPMP serves several purposes: 1) to assist participants to identify and follow through on their personalized goals; 2) to assess treatment adherence; and 3) to cross-validate data that are collected using the Chronic Pain Coping Inventory.

#### Outcome measures

##### Primary outcome

*Roland-Morris disability questionnaire (RMDQ): *The RMDQ [50] is widely used to assess physical disability associated with low back pain. The RMDQ has been demonstrated to be valid, reliable, and responsive to change [50-55]. Although developed as a measure of physical disability related to back pain, the RMDQ, re-worded without reference to the back, has been found to be a reliable and valid measure of physical disability for patients with other chronic pain problems as well [52]. The RMDQ is scored from 0–24, with higher scores indicating more severe physical disability. Physical disability, as measured by the RMDQ, is the primary study outcome.

##### Secondary outcomes

*Brief pain inventory (BPI): *The BPI is a widely-used, reliable, valid instrument that assesses pain history, location, intensity, and activity interference [56,57]. For this study, pain intensity is measured by calculating the mean of four items in which respondents are asked to rate their average, current, least, and worst pain during the past week, using a scale of 0 ("No pain") to 10 ("Pain as bad as you can imagine"). [58].

Pain-related interference is a composite measure of the degree to which pain limits a person's general function [57]. This variable is calculated as the mean of ratings of pain interference with general activity, mood, walking, work (including housework), relations with others, sleep, and enjoyment of life. Each item is rated on a scale of 0 ("Does not interfere") to 10 ("Completely interferes").

*Geriatric depression scale (GDS): *The GDS [59] is a 30-item self-report measure specifically designed to assess depressive symptoms in older persons. Scores of 11 or higher are considered indicative of depression in older adults. Good sensitivity and specificity for detecting depression in geriatric psychiatric and medical outpatients has been demonstrated (84–100% sensitivity; 73–96% specificity) [60,61]. The GDS was selected over other available depression measures because of its screening efficiency with geriatric outpatient populations, its focus on affective rather than physical symptoms, and its true/false scoring format, which studies have found to be simpler for older adults to complete [61].

### Sample size calculations and statistical analyses

#### Power analysis/sample size calculations

A mixed effects model will be used to analyze data using the participant as the unit of analysis and controlling for baseline value of the outcome as a covariate. A reasonably accurate approximation to this analysis could be obtained by the following procedure: first compute change scores (pre to post) for each person, then collapse to get the mean change score within each site, then do t-tests on these means. This simpler model was used for power calculations, since it allows standard software to be used.

The plan to randomize by site, rather than by individual participant, required additional considerations in calculating statistical power. With this group-randomized design, power depends on the correlation of people within sites, or the *intra-class *correlation. Effect size is defined as the mean change score of all individuals in the intervention group minus the mean change score of all individuals in the control group, divided by the standard deviation of change score within groups. Power calculations for the proposed study are based on estimates of 34 sites (17 intervention and 17 control), 6.4 participants per site (N = 218) providing data at 12 months (20% attrition rate).

Table 3 shows how power (the probability of detecting a difference) varies with the correlation of individuals within site and the effect size. The second column of this table shows the "effective sample size," meaning that the study would have the same power as a study with this sample size and no clustering. If the correlation is zero, the effective sample size is 272, the actual sample size. A correlation of 1 would indicate that all individuals in each site have exactly the same outcome (i.e., no different from having one person per site), so the effective sample size would be 27. Analysis of data from a pilot study showed an intra-class correlation (ICC) of .07 [62]. Although this estimate should be interpreted cautiously because of the limited number of sites in the pilot study, it indicates that the intra-class correlation will probably be fairly small, perhaps 0.05 to 0.1. Our target sample size of 273 enrolled and 20% attrition at 12-month follow-up (yielding a final sample size of 218), assuming ICC=.1, will result in 84% power for detecting an effect size of .5, which Cohen [63] refers to as a "moderate" effect size.

**Table 3:** Power for detecting a difference between the intervention and control group, depending on effect size and intra-class correlation (34 sites, average 6.4 subjects per site)

Intra-class correlation	Effective sample size	Effect size (the difference in means between the two groups, divided by the within-group standard deviation)			
		.40	.50	.60	.70
0.00	218	**84%**	**96%**	**99%**	**100%**
0.05	171	74%	**90%**	**97%**	**100%**
0.10	141	65%	**84%**	**94%**	**98%**
0.20	105	52%	71%	**86%**	**94%**
0.30	83	43%	61%	77%	**88%**
0.50	59	32%	46%	61%	75%
1.00	34	20%	29%	40%	51%

#### Statistical analysis

The test of hypothesis 1 compares the SMG and BOOK participants on the primary outcome (physical disability) and secondary outcomes (pain intensity, pain-related interference with activities, and depressive symptom severity) at each of the 3 follow-up assessments. The analytic method that we will use to evaluate this hypothesis is the mixed effects analysis of covariance (ANCOVA), as proposed by Laird and Ware [64] and implemented in the SAS PROC MIXED procedure [65,66]. This model will have two random effects, *site *and *person nested within site*. *Group *(i.e., treatment or control) and *Time *will be fixed effects. The repeated measurements of physical disability at post-intervention, 6 months, and 1 year will be the outcome measure. The baseline value of physical disability will be included in the model as a covariate. Any baseline variables that are correlated with the outcome variable and/or differ between the two treatment groups (e.g., gender, age, comorbidity) will also be included as covariates in the analysis. If the main effect for *group *is significant, contrasts within this model will be used to test for treatment effect separately at each of the three outcome times. Secondary analyses will be similar, fitting a mixed effects model that uses one of the secondary outcomes (e.g., pain intensity, pain-related interference, and depression) in place of physical disability. The analysis of Hypothesis 1 will be by intent-to-treat.

Hypothesis 2 involves comparing the SMG and BOOK groups on changes in process variables (pain-related beliefs and coping strategies). A mixed effects model, as described under hypothesis 1, will be used for these analyses. As for hypothesis 1, baseline variables that are predictive of outcome and/or differ between groups will be included as covariates in the analyses for hypothesis 2.

Hypothesis 3 involves the correlation of changes in beliefs and coping to changes in the outcome variables. For each assessment time, change from baseline will be computed and scatter plots will be used to describe relationships, with Pearson and/or Spearman correlation coefficients used to summarize the strength of the association. Although we hypothesize that significant associations in changes will occur only in the SMG group, we also will perform exploratory analyses in the BOOK group to assess for these associations.

In addition to performing major analyses to test study hypotheses, we will also perform exploratory analyses to examine whether there are subgroups of participants in whom the intervention had a particularly strong or a particularly weak effect. For example, we will explore whether: (1) there is a difference in response to therapy based on age group (young-old, mid-old, old-old); (2) men respond differently to therapy than women; and (3) pain severity at baseline is related to strength of treatment effect. These analyses will be performed using the ANCOVA described above, augmented by adding, for example, an indicator for female gender and the interaction term between gender and treatment group.

## Discussion

Persistent pain is a common problem in older adults that can be debilitating. Self-management strategies that incorporate physical and psychosocial pain coping skills are effective in decreasing pain and improving function and mood in younger adults. Little is known, however, about the efficacy of this therapy for older adults, especially those in the mid-old to old-old range. Our randomized controlled trial assesses the efficacy of such a treatment program, as compared with a control condition, in decreasing pain and improving physical and psychosocial functioning in elderly retirement community residents with chronic pain.

## Competing interests

None declared.

## Authors' contributions

ME and JAT developed the intervention and conducted the pilot test of the self-management groups. KCC developed the analysis plan, conducted the power calculations, and wrote the related sections of the paper. CAK assisted in refining the intervention. ME and JAT wrote the initial description of the intervention and this article. All authors read and approved the final manuscript.

## Pre-publication history

The pre-publication history for this paper can be accessed here:


